# Empowering Pharmacists in Type 2 Diabetes Care: Opportunities for Prevention, Counseling, and Therapeutic Optimization

**DOI:** 10.3390/jcm14113822

**Published:** 2025-05-29

**Authors:** Sarah Uddin, Mathias Sanchez Machado, Bayan Alshahrouri, Jose I. Echeverri, Mario C. Rico, Ajay D. Rao, Charles Ruchalski, Carlos A. Barrero

**Affiliations:** 1 Department of Pharmaceutical Sciences, School of Pharmacy, Temple University, Philadelphia, PA 19140, USA; sarah.uddin@temple.edu (S.U.); mathias.sanchez@temple.edu (M.S.M.); bayan.alshahrouri@temple.edu (B.A.); joseignacioeche@gmail.com (J.I.E.); mario.rico@temple.edu (M.C.R.); 2 Center for Metabolic Disease Research, Lewis Katz School of Medicine, Temple University, Philadelphia, PA 19140, USA; ajay.rao@temple.edu; 3 Department of Pharmaceutical Practice, School of Pharmacy, Temple University, Philadelphia, PA 19140, USA

**Keywords:** diabetes mellitus, type 2 diabetes, disease management, pharmacists, intervention, preventive medicine

## Abstract

Diabetes is a growing chronic disease with complications that impose a significant burden on healthcare systems worldwide. Pharmacists are readily accessible for diabetes management beyond simply dispensing medications. Consequently, they are involved in disease prevention and detection, therapy management, and patient monitoring. However, with the current escalating impact of diabetes, pharmacists must upgrade their strategies by integrating guidelines from sources like the American Diabetes Association (ADA) 2024 with pharmacy expertise. This perspective serves as a guide for pharmacists, identifying key foundations involved in diabetes management, highlighting five crucial steps for optimal disease control, ranging from prevention strategies to pharmacist-led counseling interventions. We employed PubMed, CDC, WHO guidelines, and key reference texts. Searches were performed using combinations of terms such as “pharmacist”, “type 2 diabetes”, “diabetes prevention”, “pharmacist intervention”, and “diabetes management”, covering publications from January 2010 to March 2025. Studies were included if they focused on pharmacist-led prevention, intervention, or management strategies related to type 2 diabetes (T2D) and were published in English. Studies focusing exclusively on type 1 diabetes were excluded. Generative artificial intelligence was employed to order and structure information as described in the acknowledgments. Conflicting evidence was resolved by giving relevance to recent systematic reviews, randomized trials, and major guidelines. Additional insights were gained through consultations with PharmD professionals experienced in diabetes care. Evidence from selected studies suggests that pharmacist-led care models may enhance and promote the early detection of T2D, improve therapy adherence, enhance glycemic control, and increase overall treatment efficiency. This work suggests that pharmacists must play a key role in diagnosing, preventing, managing, and mitigating the consequences associated with T2D. They must contribute to early treatments with appropriate training and involvement to improve therapeutic outcomes and reduce diabetes-related complications.

## 1. Introduction

Diabetes is a multifaceted disorder characterized by deficits in carbohydrate metabolism that compromise insulin’s function in tissues and lead to elevated blood glucose levels [1]. Diabetes is a highly prevalent and costly condition, with approximately one in ten Americans living with the disease [2,3], and one in four are never diagnosed; in fact, the diagnosis of T2D may be delayed by six to thirteen years after the disease onset due to asymptomatic progression and lack of pursuit of preventive medicine [4,5]. For these reasons, diabetes is commonly referred to as a silent killer. It can progress for years without noticeable symptoms, resulting in serious complications such as cardiovascular disease, kidney disease, and peripheral neuropathy. Prediabetes, the intermediate state between normoglycemia and diabetes, is estimated to be present in one out of every three adults in the United States, increasing the risk of developing diabetes, additional cardiovascular diseases, and other cardiometabolic outcomes [6].

The pathophysiology of diabetes stems from a key hormone, insulin, produced by the β-cells in the pancreas, that helps the body utilize available glucose efficiently via the glucose transport-4 receptor (GLUT4). Insulin targets the cells to take glucose inside through a signaling cascade downstream of insulin receptor activation. Insulin also signals the liver to increase sugar storage and/or triggers other cells to take in glucose to decrease the amount in the bloodstream [7]. Diabetes is either caused by the pancreas’s inability to produce insulin or the body’s failure to utilize insulin effectively [8]. When insulin signaling is disrupted due to insufficient production or cellular resistance, glucose uptake by the muscles and liver becomes impaired. As a result, chronic hyperglycemia develops, which is presumed to induce epigenetic changes, protein glycosylation, overexpression of growth factors, and the production of mitochondrial-derived reactive oxygen species (ROS), ultimately triggering inflammation [9].

The ADA classifies diabetes into four categories: (1) type 1 diabetes (T1D), caused by autoimmune β-cell destruction; (2) T2D, involving progressive β-cell dysfunction with or without insulin resistance; (3) diabetes resulting from genetic mutations, exocrine pancreatic diseases, or drug-induced effects; and (4) gestational diabetes (GDM) [10]. TD2 remains the most prevalent type of diabetes. Approximately 95% of individuals with diabetes have T2D, whether or not they are also resistant to insulin.

T2D is characterized by a relative deficiency of insulin, which ultimately results in progressive hyperglycemia. This condition often presents without symptoms and is frequently undiagnosed during the initial stages of the disease. T2D has a substantially heritable characteristic, subsequently influenced by environmental risk factors that include but are not limited to obesity and sedentary lifestyles [11]. Due to its gradual onset, T2D often remains undiagnosed for many years. By the time of diagnosis, around 30% of patients may already have severe complications like cardiovascular disease (such as myocardial infarction and stroke), nephropathy leading to chronic kidney disease, retinopathy causing vision impairment or blindness, and peripheral neuropathy, increasing the risk of foot ulcers and amputations [12,13,14].

Prediabetes, the intermediate state between normoglycemia and diabetes, is estimated to be present in one out of every three adults in the United States, increasing the risk of developing diabetes, additional cardiovascular diseases, and other cardiometabolic outcomes [6].

Metabolic syndrome (MetS) is a cluster of metabolic dysregulations, including insulin resistance, atherogenic dyslipidemia, central obesity, and hypertension, that increases the patient’s risk of stroke, heart conditions, and often diabetes [15]. A common way by which patients can develop MetS is by being unable to control excess weight, whether due to overeating, sedentary behavior, and/or genetic predisposition, leading to potential insulin resistance and eventually hyperglycemia, resulting in diabetes [16].

MetS is linked to insulin resistance, which plays a central role in the patient’s pathophysiology. Insulin resistance triggers metabolic dysregulation, increasing the risk of T2D and atherosclerotic cardiovascular disease (ASCVD). In addition, MetS is often associated with chronic inflammation and a prothrombotic state, which causes vascular injury and further exacerbates metabolic dysregulation. Due to the significant detrimental impacts on health, early detection and lifestyle modification interventions (e.g., regular health checks, exercise, and proper nutrition) are crucial for slowing diabetes progression and achieving better long-term outcomes [17,18].

According to the United States Bureau of Labor Statistics, the pharmacist’s primary function is to deliver prescription medications to patients and provide information on the drugs and their use [7]. However, their responsibilities extend beyond that. A pharmacist in the United States must complete a clinical degree in a PharmD program that lasts over four years and encompasses biomedical, clinical, pharmaceutical, administrative, social, and behavioral sciences. They are medication experts and the most accessible healthcare providers, particularly in primary care settings. The widespread adoption of integrative medicine positions pharmacists as essential links within the healthcare team. This encourages them to move beyond their traditional role as drug dispensers. They become integrated healthcare members who provide therapeutic recommendations, offer evidence-based drug information to the interprofessional team, and conduct medication reconciliation and patient education [11].

A thorough literature review identified that pharmacist interventions in patients with T2D have been shown to improve HbA1c levels, reduce other associated complications, and enhance economic outcomes. As a result, pharmacists can optimize pharmacological therapy for their patients with diabetes. Given their accessibility, pharmacists are invaluable in managing care for diabetic patients, resulting in improved health outcomes [19]. Hence, this article will present a systematic process for pharmacists to enhance care for patients with diabetes.

## 2. Diagnosis

Patients are diagnosed with prediabetes or T2D based on plasma glucose criteria. Tests used for diagnosis include (1) random or fasting plasma glucose (FPG), (2) the oral glucose tolerance test (OGTT), a 2 h plasma glucose (2 h PG) value after 75 g oral glucose consumption, and (3) the hemoglobin A1C (HbA1C) test [1,20]. According to the ADA, no diagnostic test is universally preferred, and it advises confirming abnormal results with a second test unless clear hyperglycemia and symptoms are present [21]. FPG and HbA1c are commonly used due to their convenience and reproducibility, whereas OGTT, although more sensitive for detecting early glucose abnormalities, is less practical in routine settings. FPG has high specificity, and HbA1c, despite its ease of use, may miss early cases and is affected by hematologic conditions [22].

### 2.1. Prediabetes

“Prediabetes” means having HbA1C between 5.7 and 6.4% or FPG levels between 100 and 125 mg/dL (between 5.6 and 6.9 mmol/L), and IGT is defined as 2 h PG levels after 75 g OGTT between 140 and 199 mg/dL (between 7.8 and 11.0 mmol/L) [1] (Table 1). Prediabetes is a term that should not be viewed as a clinical entity exclusively, but more so a term that describes patients who are at high risk for developing T2D and cardiovascular disease [23]. It is crucial to identify prediabetic patients to enable early detection, diagnosis, and subsequent treatment, thereby achieving better health outcomes [24].

### 2.2. Diabetes

To diagnose a patient with diabetes, they must attain unequivocal hyperglycemia or two positive tests from the following criteria: FPG ≥ 126 mg/dL OR 2 h PG ≥ 200 mg/dL during an OGTT (using a glucose load of 75 g anhydrous glucose in water), OR HbA1C ≥ 6.5%, OR random plasma glucose ≥ 200 mg/dL (in patients with symptoms of hyperglycemia such as polyuria, polydipsia, and polyphagia) [1] (Table 1).

### 2.3. Metabolic Syndrome (MetS)

MetS is diagnosed when an individual exhibits at least three criteria: FPG, abdominal obesity, hypertriglyceridemia, low HDL cholesterol, or hypertension. These criteria were established by the National Cholesterol Education Program Adult Treatment Panel III (NCEP ATP III) and reaffirmed by the American Heart Association (AHA) and the International Diabetes Federation (IDF). In detail, the diagnosis of MetS requires three of the following criteria: FPG ≥ 100 mg/dL (5.6 mmol/L) or a diabetes diagnosis; waist circumference thresholds differ by ethnicity, but typically, values exceeding 102 cm (40 inches) in men and 88 cm (35 inches) in women indicate abdominal obesity; triglycerides ≥ 150 mg/dL (1.7 mmol/L); HDL cholesterol < 40 mg/dL in men and <50 mg/dL in women; or blood pressure ≥ 130/85 mmHg [18] (Table 1 and Table 2).

### 2.4. Screening for Diabetes and Prediabetes

While mortality rates from hypertension and dyslipidemia have decreased over the years, diabetes and overweight issues have not [25]. Despite the high incidence of diabetes, the CDC reports that up to 21.4% of the diabetic population consider themselves asymptomatic or do not acknowledge having the condition. In contrast, only 15.3% of prediabetics have been informed of their condition [26]. Screening asymptomatic adults with diabetes yields ambiguous results. Although these studies may improve health outcomes through earlier identification, diagnosis, and treatment, the evidence to support this claim remains controversial [27]. Simultaneous screening for diabetes and prediabetes has not yet been evaluated in randomized trials [27].

Screening asymptomatic adults with diabetes yields ambiguous results. Although these studies may improve health outcomes through earlier identification, diagnosis, and treatment, they report little direct evidence to support this claim. Furthermore, simultaneous screening for diabetes and prediabetes has not yet been tested in randomized trials [27].

The United States Preventive Services Task Force (USPSTF) recommends screening for diabetes in asymptomatic, non-pregnant adults aged 35 to 70 who have a BMI ≥ 25 or higher, with a recommended interval of 3 years [26]. Although diagnostic tests for diabetes can be used for screening, the most recommended test is the HbA1c, as it provides an average of the glycemic profile from the last 3 months.

Pharmacists’ implementation of validated clinical tools such as the ADA diabetes risk test may be beneficial in identifying individuals who require a diagnostic test, especially overweight or obese individuals. Once completed, the pharmacist may redirect the patient for medical evaluation to further evaluate the diagnosis and/or potential treatment.

Pharmacists can enhance diabetes screening efforts by integrating digital platforms and community outreach initiatives. Digital tools like mobile health applications, risk assessment calculators, and telehealth services enable pharmacists to identify high-risk individuals and provide targeted follow-up recommendations remotely. Additionally, community outreach programs, including health fairs, workplace wellness events, and partnerships with local organizations, can expand access to screening, particularly in underserved populations [28]. By leveraging technology and community engagement, pharmacists can play a pivotal role in improving early detection rates for prediabetes and T2D.

## 3. Pharmacists in Diabetes Management

Pharmacists play a key role in supporting patients with adherence to treatment regimens, particularly in chronic disease management. Pharmacist-led screening programs have been shown to enhance the detection of MetS or its components, facilitating early intervention and management by maintaining communication with physicians [29]. Additionally, pharmacists may promote glucometer testing or at-home diabetes testing, which can help patients identify potential prediabetic or diabetic symptoms and recommend physician referrals in appropriate situations [30]. It is essential to note that training will be required to effectively apply these tools, including understanding scoring systems, communicating results, and knowing when to refer to a physician.

Furthermore, the procedures associated with caring for patients with chronic diseases require pharmacists to take on an educational role with patients, providing directed education about TD2. Pharmacists can facilitate patients’ understanding of diabetes and its associated complications, particularly for those experiencing challenges with treatment adherence. They might educate patients on maintaining their daily routines, promoting medication adherence, and making lifestyle changes that support their goals while informing them about nonadherence’s consequences. Educating patients in a manner that acknowledges their dilemma can additionally provide emotional support for patients who feel the burden of their experience [31,32].

To improve the management of patients with prediabetes or diabetes, pharmacists may implement structured protocols that guide clinical and behavioral interventions. This approach allows pharmacists to guide patients through five key steps [33]:

(1) Adhering to a balanced diet;
(2) Practicing physical activity;
(3) Providing proper mental health management;
(4) Evaluating pharmacological options and conducting audits on medical interventions;
(5) Addressing the complications of diabetes.

The initial three stages of the pharmacist’s five-step process encompass necessary lifestyle modifications regarding diet, exercise, and mental health as part of a strategy to improve diabetes management and mitigate the risk of complications. The final two stages prompt the pharmacist to optimize medication and collaborate critically with the healthcare team in decision-making.

### 3.1. Adhering to a Balanced Diet

To begin, the patient needs to be informed about the importance of following a balanced hypocaloric diet and to understand the potential effects of a poor diet on developing diabetes or diabetes-related complications. Pharmacists can inform patients that diabetes occurs when sugars are not properly stored in the blood, preventing the normal regulation of the body’s natural insulin. Thus, a rich diet with sugar, fats, and other glucose-rich substances can predispose a patient to becoming diabetic or exacerbating existing diabetes. Therefore, it is important to emphasize the role of lifestyle interventions in improving glycemic control and reducing the risk of complications [33].

The growing incidence of diabetes in the United States has been associated with rising obesity rates, which are driven by caloric intake and physical inactivity [34]. Pharmacists can play a crucial role in educating patients about maintaining a balanced diet and how adopting healthier eating habits can help reduce the risk of diabetes. Pharmacists must explain to each patient what those lifestyle changes will entail. For example, the definitions of a “balanced diet” and “150 min of exercise” can differ from patient to patient. Thus, the pharmacist can help the patient contribute to what a “balanced diet” and “150 min of exercise” mean for them, thereby optimizing lifestyle changes tailored to each individual. In this section, we will discuss various diets recommended for diabetic patients and suggest possible lifestyle changes they might consider for their lifestyle [31]. Pharmacists should also refer patients to registered dietitians and/or nutritionists for counseling and ensure at least one follow-up appointment within the first three months following a diabetes diagnosis [25].

The following two practices have proven great success in controlling and preventing complications for patients diagnosed with diabetes.

#### 3.1.1. DASH Diet

The DASH (Dietary Approaches to Stop Hypertension) dietary plan is suitable for individuals with diabetes. The DASH dietary pattern is linked to improvements in blood pressure control, insulin resistance, hyperlipidemia, and obesity. This dietary approach promotes foods low in saturated fat, total fat, cholesterol, and sodium while being rich in potassium, calcium, magnesium, fiber, and protein. Individuals following the DASH plan are encouraged to consume whole grains, fat-free or low-fat dairy products, fruits, vegetables, poultry, fish, and nuts. Foods to restrict include fatty meats, full-fat dairy products, tropical oils (e.g., coconut, palm, and palm kernel), sweets, and sugar-sweetened beverages [35]. In a randomized crossover trial, the DASH diet improved blood lipids and blood pressure while decreasing HbA1C by 1.7% and fasting blood glucose levels by 29%. Although this diet is not specifically designed for direct weight loss, its emphasis on higher consumption of fruits and vegetables and lower-fat dairy products and reduced consumption of red meat and sweets can be beneficial for those looking to lose or maintain weight [36]. However, it is recommended that normotensive patients maintain regular sodium intake, as 23 clinical trials have shown that low sodium consumption in diabetic patients may hinder their glycemic targets due to an increase in stress hormones such as noradrenaline, aldosterone, renin, and angiotensin-II [37]. Another important consideration is that while fruit consumption is encouraged to improve the glycemic targets of diabetic patients, it is crucial to remember that fruits are counted as carbohydrates, which need to be integrated into their meal plan [38].

#### 3.1.2. The Plate Method—CDC

**Figure 1:**
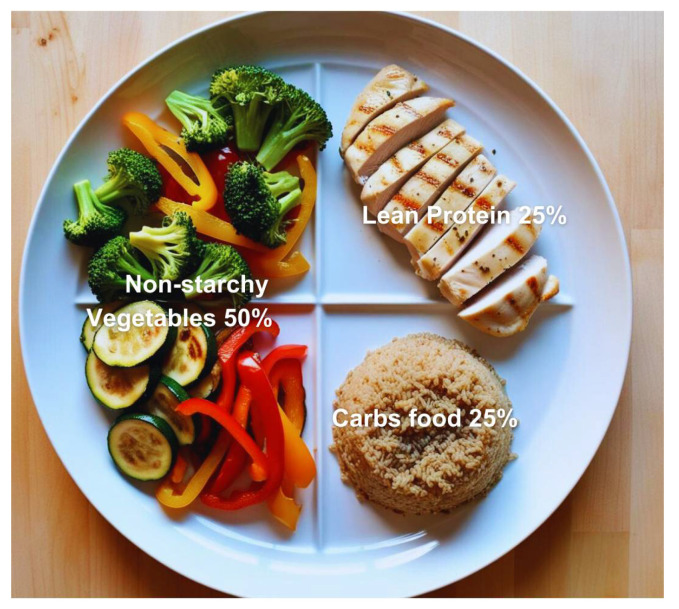
The Plate Method illustrates a visual approach to balanced eating for managing diabetes. A plate is divided into three compartments, with half of the plate designated for non-starchy vegetables (leafy greens, broccoli, and peppers), one quarter of the plate for lean protein (chicken, fish, tofu, or eggs), and one quarter of the plate for carbohydrates (whole grains, starchy vegetables, or legumes). The Plate Method encourages a balanced intake of nutrients and portion control, which involves following dietary recommendations to help manage blood glucose levels. A small serving of healthy fats and water, or low-calorie beverages, is also recommended as part of the meal. Image generated and formatted using Canva (www.canva.com, accessed on 16 March 2025), a graphic design platform.

The Plate Method (Figure 1) is a dietary approach that utilizes the actual plate to guide healthier eating habits. This method enables patients to easily manage their macronutrients without adhering to a strict diet plan. The Plate Method involves filling half of one’s 9-inch dinner plate (about the length of a business envelope) with non-starchy vegetables, such as salad, green beans, broccoli, cauliflower, cabbage, and carrots. One-quarter of the plate will contain lean protein, such as chicken, turkey, beans, tofu, or eggs. The remaining quarter will consist of carb-rich foods, including grains, starchy vegetables (such as potatoes and peas), rice, pasta, beans, fruit, and yogurt. A cup of milk also counts as a source of carbohydrates. This method is a practical and adaptable approach that can be used to counsel patients seeking ways to manage their diabetes [39,40]. The Plate Method is widely regarded as a valuable and easy-to-understand dietary tool; studies have shown that patients find it highly usable, with improved portion control, meal planning confidence, and dietary adherence. A randomized controlled trial by Cavanaugh et al. (2016) demonstrated that using the Plate Method in diabetes self-management education significantly improved glycemic control, particularly among patients with lower numeracy skills [40,41].

In addition to advising patients to adhere to the recommended diet, it is equally important to encourage them to look into their diet from the outset. The nutrition-related diets and practices mentioned above enable the pharmacist to counsel patients on healthier eating habits that have been shown to enhance diabetes management, allowing patients to incorporate aspects of these diets into their eating plans.

### 3.2. Practicing Proper Physical Activity

Any physical activity induces muscle glucose uptake through non-insulin-dependent pathways while improving systemic and possibly hepatic insulin sensitivity, which can last up to 72 h, depending on the duration and intensity of the exercise performed [42]. However, higher levels of exercise intensity are associated with more substantial improvements in HbA1C and cardiorespiratory fitness. The American Diabetes Association recommends at least 150 min of moderate- to vigorous-intensity, combined aerobic and resistance physical activity each week, with no more than two consecutive days without activity. If this goal cannot be met, engaging in walking, yoga, housework, gardening, swimming, and dancing can help prevent sedentarism, such as interrupting continuous sitting for longer than 30 min [43]. However, the intensity of 150 min of weekly physical activity may vary among individuals. To qualify as moderate-intensity exercise, physical activity should raise the heart rate to 50–70% of an individual’s maximum heart rate (calculated as 220 minus age). For vigorous-intensity exercise, heart rates should reach 70–85% of the maximum [44,45,46]. Patients can engage in various activities, including weight training, aerobic exercise, jogging, dancing, swimming, and dog walking, among others. A specific step goal helps patients track and assess their daily physical activity [47]. The ADA recommends aiming for 10,000 steps per day to help reduce the risk of type 2 diabetes (T2D). Pharmacists can help patients start or maintain adequate physical activity that aligns with their schedules, needs, and restrictions [48]. Patients need to pay special attention to hypoglycemia risk assessment, especially for those on insulin treatment and following weight loss surgery [42].

### 3.3. Providing Proper Mental Health Management

In current practice, mental health is often not prioritized when creating a care plan for a patient. Mental health is usually neglected in favor of other aspects of the patient that are allegedly more important. Some fail to realize that when a patient is anxious, depressed, aggravated, or afflicted, their problems go beyond emotional and can translate to physical sickness. One of the most critical patient populations worth promoting these mental health interventions to is those who are prediabetic. If provided preventative measures of mental health interventions were taken, these patients could potentially prevent the later occurrence of diabetes [49,50,51].

Depression and anxiety are notably prevalent among individuals with T2D. A 2023 study reported that approximately 29.2% of U.S. adults with diabetes experience depression, a rate significantly higher than the 17.9% observed in adults without diabetes [52]. Moreover, a meta-analysis published in 2021 reported that interventions aimed at treating depression in patients with T2D significantly improved glycemic control, with an effect size of d = 0.208 (95% CI: 0.088–0.329; *p* = 0.001) [53]. Recognizing the impact of mental health on diabetes management, the ADA recommends routine psychosocial screening for depression, anxiety, diabetes distress, and disordered eating at the time of diagnosis and periodically thereafter [1].

Poor mental health itself can cause a myriad of ailments that lead to diabetes or its risk factors. Stress and emotional distress can lead to unhealthy habits of binge eating, decreased motivation, and reliance on drugs and alcohol, all of which may lead to a lack of physical activity, putting the patients at risk of developing T2D. Chronic stress alone can lead to elevated blood sugar levels. Moreover, unmanaged mental health issues can lead to nonadherence to one’s diabetes treatment plan [54]. Untreated mental health issues can worsen diabetes, and complications with one’s diabetes can worsen mental health [55]. Hence, one key method to prevent diabetes and avert complications is to promote adequate mental health, which involves promoting mental well-being by encouraging healthy psychological practices and ensuring the early detection of mental health issues.

Pharmacists can conduct brief mental health screenings to identify individuals who may be at greater risk of developing mental health disorders (e.g., family history, substance abuse, traumatic life events, cultural factors, sleep problems, etc.). If necessary, pharmacists can refer these patients to mental health professionals, therapists, community resources, support groups, and external educational programs that focus on enhancing mental health. Recommended self-care strategies may include maintaining a consistent sleep schedule of 7–9 h per night, engaging in mindfulness practices such as journaling and meditation, limiting exposure to social media, incorporating regular reading, participating in enriching hobbies, and fostering social connections through meaningful time spent with family and friends [56].

Studies show that therapy and lifestyle modifications are often enough to support patients’ mental health. However, medication is usually needed for higher efficacy, and the pharmacist can assist with their knowledge and counseling [57,58]. Developing a therapeutic care plan must consider the patient’s mental health as a vital element. Neglecting mental health in patients with or at risk of diabetes may contribute to reduced self-care, treatment adherence, and overall disease management.

### 3.4. Pharmacological Treatments

Pharmacological treatments for patients diagnosed with T2D should begin at diagnosis and must always be accompanied by healthy lifestyle behaviors that focus on a balanced diet, regular exercise, and effective weight management. This approach ensures that pharmacological interventions are maximally effective. Rather than selecting a first-line treatment, a person-centered approach to diabetes treatment is warranted. To address the complexity of T2D, therapy should consider comorbidities (particularly cardiovascular, renal, and hepatic), weight management goals, glycemic control, and the patient’s monetary preferences [59]. Although many pharmacotherapy options exist, the role of pharmacists extends beyond simply choosing a medication. It not only includes documenting medication reconciliation to identify duplicates or potential interactions but also involves providing patient-centered titration for individualized efficacy and safety, as well as deprescribing to minimize the risk of adverse drug events and unnecessary polypharmacy. In addition, pharmacists play a critical role in adherence management through the optimization of side effects, the development of simplified dosing strategies, and education to ensure understanding. Through collaborative practice agreements (CPAs), pharmacists aim to collaborate with providers to adjust medications according to patient-specific outcomes, enhancing the therapeutic effectiveness of pharmacotherapy while minimizing potential risks [60,61,62].

#### 3.4.1. Metformin

Metformin remains an essential drug in managing glycemic control; however, according to the ADA 2025 guidelines, a more individualized approach is warranted. In specific populations, such as those with existing cardiovascular disease, with or without chronic kidney disease, or with obesity, GLP-1 receptor agonists and SGLT2 inhibitors may be prioritized as initial therapy due to demonstrated benefits in these groups [59].

Metformin is a well-tolerated drug that is effective both as a monotherapy and in combination with other glucose-lowering medications. It has a long-standing track record for safety and efficacy, is inexpensive, and may reduce the risk of cardiovascular events and death [59,63]. Adverse effects associated with metformin therapy typically relate to gastrointestinal intolerance, rare cases of lactic acidosis in patients with a glomerular filtration rate (GFR) below 30 mL/min/1.73 m^2^, and B12 deficiency that can worsen neuropathic symptoms [64,65]. It is essential to note that all pharmacological therapy should be initiated in conjunction with counseling that emphasizes lifestyle modifications, including a balanced diet and regular exercise.

The treatment should be tailored to the clinical needs and comorbidities of the patient [66]. Other pharmacological therapies that are available for managing T2D in addition to metformin include the following.

#### 3.4.2. DPP-4 Inhibitors

Dipeptidyl peptidase-4 inhibitors, such as sitagliptin (Januvia) and linagliptin (Tradjenta), work by inhibiting the DPP-4 enzyme, thereby prolonging the action of incretin hormones. These drugs improve post-meal glucose control and are generally well-tolerated, though they are less effective in lowering HbA1C than GLP-1 receptor agonists [67,68].

#### 3.4.3. SGLT2 Inhibitors

Sodium-glucose co-transporter-2 (SGLT2) inhibitors, including empagliflozin (Jardiance) and canagliflozin (Invokana), lower blood glucose levels by enhancing glucose excretion in urine. Furthermore, they provide cardiovascular and renal protection, making them a suitable option for patients with heart failure or chronic kidney disease. Renal function may inhibit SGLT2 when GFR < 40 mL/min/1.73 m^2^ [69,70,71].

#### 3.4.4. Thiazolidinediones (TZDs)

Thiazolidinediones (TZDs), such as pioglitazone (Actos), promote insulin sensitivity by binding to adipose and muscle tissue. While TZDs can lower HbA1C, they may also cause weight gain, fluid retention, and an increased risk of fractures, which can restrict their use in particular [72]. Because of the potential for fluid overload, the use of TZDs should be limited in patients with heart failure and kidney disease [73].

#### 3.4.5. Sulfonylureas

Sulfonylureas, namely glyburide and glipizide, function to stimulate the pancreas to secrete insulin in order to lower blood glucose levels. These medications are highly effective and widely used, primarily due to their low cost. However, they carry risks of causing weight gain and hypoglycemia, which can make it less favorable for many patients [74]. Addressing the adverse side effects associated with sulfonylureas can help prevent complications and thus improve adherence.

#### 3.4.6. Alpha-Glucosidase Inhibitors

Alpha-glucosidase inhibitors, such as acarbose (Precose), slow the breakdown and absorption of carbohydrates in the intestines, reducing post-meal glucose spikes. While practical, these drugs are less commonly used due to gastrointestinal side effects such as bloating and diarrhea [75].

#### 3.4.7. Bile Acid Sequestrants

Drugs like colesevelam (Welchol) moderately lower blood glucose levels while also reducing LDL cholesterol. Due to their limited glucose-lowering capacity, they are generally well tolerated but are not typically regarded as first-line agents [76].

#### 3.4.8. Combination Therapies

Combination therapies are especially advantageous for patients with T2D who do not meet glycemic targets with monotherapy, those with existing cardiovascular or renal disease, or individuals with elevated baseline HbA1c levels (>9%). However, combination treatments come with potential risks, such as an increased likelihood of hypoglycemia (mainly when insulin or sulfonylureas are used), higher medication costs, and difficulties with adherence due to complex regimens. Customizing combinations based on patient-specific factors like comorbidities, socioeconomic status, and lifestyle is essential to maximize benefits and reduce risks [77]. Fixed-dose combination therapies, such as metformin plus sitagliptin (Janumet) or metformin plus empagliflozin (Synjardy), can simplify treatment and improve adherence by reducing pill burden. These combinations can be customized based on the patient’s glycemic needs and comorbidities [78].

#### 3.4.9. GLP-1 Receptor Agonists

Glucagon-like peptide-1 receptor agonists, a class of medications that comprise Ozempic (semaglutide) and Trulicity (dulaglutide), enhance insulin release, inhibit glucagon release, delay gastric emptying, and decrease appetite. These medications have demonstrated significant cardiovascular benefits in patients at risk for cardiovascular disease. Furthermore, they promote weight loss, making them a good choice for patients with obesity and diabetes [79,80,81].

#### 3.4.10. Dual GLP-1 and GIP Receptor Agonist

Tirzepatide, similar to semaglutide and dulaglutide, is a GLP-1 receptor agonist that, in addition to the effects described for GLP-1RAs, offers an even more significant weight-lowering impact due to its dual action as a GIP receptor agonist, which encourages fat breakdown and decreases fat accumulation [82,83].

#### 3.4.11. Insulin Therapy

For patients with advanced diabetes or those who are unable to attain glycemic targets with oral medications, insulin therapy becomes essential. Insulin initiation should always be considered if symptoms of hyperglycemia are present and when HbA1c levels exceed 10% or blood glucose levels are >300mg/dL [59]. Long-acting basal insulins such as glargine (Lantus) are frequently used as a starting point, with rapid-acting insulins added to control post-meal glucose spikes [84]. If glycemic goals are not achieved, or if signs of overbasalization are evident (bedtime-to-morning glucose differential over 50 mg/dL, hypoglycemia, and significant glucose variability), progression to combination injectable therapy is generally recommended, considering GLP-1 receptor agonists with dual GIP and GLP-1 RA, or dual insulin therapy. All individuals using insulin should be educated on proper administration techniques, hypoglycemia awareness, and glucose self-monitoring. Insulin therapy should never be labeled as a treatment or a sign of treatment failure to the patient [59].

#### 3.4.12. Hypoglycemia Education

Insulin and sulfonylureas can likely cause hypoglycemia, a profound adverse effect that increases the risk of heart disease, may potentially lead to brain damage from repeated severe episodes, and can even result in death. Fasting, delayed meals, physical activity, and illness can trigger hypoglycemia. Clinical manifestations include tremors, agitation, disorientation, palpitations, diaphoresis, and hunger. Patient education on self-monitoring of blood glucose, abstaining from driving with symptomatic hypoglycemia, and recommendations for initial management should be considered to prevent hypoglycemia. These medications should be avoided in the presence of hypoglycemia, whether symptomatic or with blood glucose < 70 mg/dL. The initial treatment for hypoglycemia in a conscious individual is glucose, although any carbohydrate-containing food or beverage can be used. Foods or drinks high in fat and/or protein should be avoided. This should be repeated if hypoglycemia persists fifteen minutes after the initial treatment [26].

### 3.5. Audit and Feedback on Medical Interventions

Pharmacists, as the most available drug experts in the community, are responsible for including activities towards detecting, assessing, understanding, and preventing possible drug-related problems, thus applying pharmacovigilance in their daily practice [85]. Reporting adverse drug reactions (ADRs) is pivotal in the application of pharmacovigilance since some ADRs are only discovered after marketing, especially among new medications [85]. However, a survey conducted on professional pharmacists from Portugal in 2022 has shown that less than 50% of the responders had never reported an ARD, primarily due to unawareness of ARDs, uncertainty on drug–event causality, and lack of time [86,87].

Polypharmacy, especially among the elderly, is another problem that should be addressed by pharmacists regardless of their working setting; a meta-analysis published in 2022 showed that up to 50% (95% CI 37–63%) of diabetic patients may meet polypharmacy criteria, defined as the use of five or more medicines, which has shown to negatively influence diabetic-specific and other health-related outcomes [88,89]. A randomized controlled trial (RCT) conducted in Australia has demonstrated that pharmacist-led medical review through deprescription algorithms among older people living in residential elderly care facilities yields deprescription recommendations in 28% of cases due to inappropriate prescriptions, 17% due to adverse effects or interactions, and 42% due to lack of indication. Of importance, over 70% of the pharmacists’ recommendations were accepted by the general practitioner and the medical team [90].

Despite the importance of diabetes treatment adherence, evidence has shown that it can be as low as 36% [91]. Pharmacists, as dispensing drug professionals, have the potential to detect and act on medication nonadherence, which is highly prevalent and known to be associated with therapeutic inertia and medication-related problems. Medication adherence can be assessed using the fixed medication possession ratio of medication refills and medication adherence questionnaires like the Morisky, Green, and Levine (MGL) adherence scale [92]. Implementing a pharmacist-led intervention protocol demonstrated positive results in addressing and managing medication nonadherence in an RCT conducted in Saudi Arabia. At 12 months of follow-up, participants in the MPIP (Medication Possession Improvement Program) group showed a significant improvement in overall adherence, with a total (composite) medication possession ratio (MPRt) mean (±SD) of 0.95 (±0.09), compared to 0.92 (±0.09) in the control group [92].

### 3.6. Addressing the Complications of Diabetes

The role of the pharmacist involves understanding the complications that diabetes introduces in the body to develop an accurate understanding of the medication, allowing for effective management of the patient’s diabetes. Recognizing these complications can motivate patients at risk or those who are prediabetic to adopt a healthier lifestyle [33]. Complications associated with diabetes ultimately lead to high glucose levels due to insulin resistance, resulting in hyperglycemia in the body. This condition can lead to harmful complications that may be classified as either vascular or nonvascular. Vascular complications can manifest in either a microvascular or macrovascular state [93].

Macrovascular complications of hyperglycemia affect the larger blood vessels in the body and pathologically stem from atherosclerosis; they can occur even before hyperglycemia develops. These complications include an increased risk of cardiovascular disease, stroke, and peripheral arterial disease (PAD) [94]. Conversely, hyperglycemia also leads to microvascular consequences for the body, impacting its smaller blood vessels. Additionally, retinopathy, nephropathy, and neuropathy should be discussed as microvascular complications of diabetes to be explained to patients [95].

#### 3.6.1. Retinopathy

Retinopathy is the most common complication of diabetes, contributing to over 10,000 new cases of blindness in the U.S. each year. Glucose glycosylates nonenzymatic proteins in the retina, which has been associated with retinal pericyte loss [96]. Retinopathy caused by diabetes can lead to blurred vision and, eventually, irreversible blindness. Intensive diabetes management has been demonstrated in extensive prospective randomized studies to prevent or delay the onset and progression of diabetic retinopathy, reduce the need for ocular surgery, and improve visual function [97]. Patients with T2D should undergo an eye exam prior to diagnosis. If there is no evidence of retinopathy during one of the annual eye exams and blood sugar levels are normal and well-controlled, then the patient can be screened every one to two years. In the presence of retinopathy, it is advisable to schedule dilated retinal examinations at least once a year. Of utmost importance in reducing the risk and delaying the progression of diabetic retinopathy is to optimize one’s glucose levels [98].

#### 3.6.2. Nephropathy

Diabetic nephropathy is the leading cause of renal failure in the United States and is a microvascular complication of diabetes in which elevated serum glucose damages the blood vessels surrounding the kidney. The pathology of the disease also increases blood pressure, further damaging the kidneys beyond just the effects of elevated blood pressure. Increased glucose levels can lead to increased glomerular basement membrane thickness, heightening the likelihood of microaneurysms and the formation of mesangial nodules (Kimmelstiel–Wilson bodies) along with other renal complications. Screening for diabetic nephropathy or microalbuminuria may be conducted annually via a 24 h urine collection or a spot urine measurement of microalbumin [99].

#### 3.6.3. Neuropathy

Diabetic peripheral neuropathy (DPN) is defined as damage to the peripheral nerves due to hyperglycemia. This peripheral nerve dysfunction can induce signs and symptoms of numbness/tingling, muscle weakness, sharp pains, etc. Patients with T2D should be assessed for DPN at diagnosis and receive a pinprick, temperature, ankle reflex, or vibration perception test annually via a tuning fork. A serious and near-life-threatening complication for diabetic patients with DPN is the potential for amputations [100]. Diabetic patients are at increased risk of not realizing they have a wound or ulcer on their feet and thus are at increased risk of having their lower extremities infected. This increased risk is due to complications of diabetic peripheral neuropathy (DPN), which in some cases can lead to enough nerve damage that the patient feels minimal sensations in their lower extremities, and peripheral arterial disease (PAD), which causes blood vessels to narrow leading to less blood being able to flow to the legs and feet. If a sudden infection arises within the lower extremities of these patients and they fail to notice due to their neuropathy-induced hyposensitivity, the progression of infection could ultimately lead to amputation [101].

#### 3.6.4. Diabetes Complication Risk Assessment

Traditional risk scores used to evaluate diabetes complications, such as the United Kingdom Prospective Diabetes Study (UKPDS) and Framingham risk scores, often focus primarily on cardiovascular outcomes. Furthermore, they may overlook patient ancestry and ethnicity, which can impact the accuracy of risk prediction [102]. Then, their primary focus is on single outcomes, particularly all-cause mortality, without offering a comprehensive assessment of other diabetes complications such as nephropathy, retinopathy, and neuropathy [103]. Finally, they do not consider novel biomarker or genotype data that may enhance risk prediction for specific complications [104].

Breakthroughs in computer science, biotechnology, genomics, and proteomics are transforming medical practice. Specifically, the use of machine learning, a subset of artificial intelligence, enables computer systems to discover patterns in data that further enhance the classification and risk evaluation of diabetes complications [105]. Modern risk engines like the BRAVO model utilize these novel techniques to analyze data from multinational, large-scale randomized clinical trials, simulating disease progression and predicting patient outcomes. The model provides risk stratification, treatment recommendations, and cost-effectiveness analysis [106]. With further validation, AI-driven models can enhance the management of diabetes complexity, which is driven by its heterogeneity. Pharmacists can utilize them in primary care to assist with individualized patient medication reviews and recommendations.

## 4. Pharmacists’ Involvement in the Split-Shared Care Model

T2D is an expensive disease. The 9th International Diabetes Federation Diabetes Atlas reported that in 2019, health expenditure in this group was USD 760 billion, with a projected growth to USD 825 billion by 2030 [107]. Approximately 17% of the total direct medical costs attributed to diabetes in the United States in 2022 were related to glucose-lowering medications and diabetes supplies [108]. A systematic review published in 2023 that included 28 randomized controlled trials showed that collaborative care between pharmacists and physicians led to a 1.2% (−2.5% to −0.15%) decrease in HbA1c, reductions in systolic and diastolic blood pressure of −6.42 mmHg (95% CI: −7.99 to −4.84), and a reduction in total cholesterol of −0.26 mmol/L (95% CI: −0.49 to −0.03). Moreover, 85% of pharmacists’ recommendations were accepted by physicians. However, although a few studies assessed cost-related outcomes, it was not possible to calculate overall cost-effectiveness due to inconsistent measurement methods and limited economic data [109].

A multi-center randomized controlled trial published in 2024 evaluated the effectiveness of a split-shared care model involving community pharmacists and physicians in reducing the economic burden for individuals living with uncontrolled T2D and polypharmacy. The intervention consisted of two in-person sessions, focusing on medication therapy management and blood glucose self-monitoring, as well as three follow-up calls that emphasized lifestyle modifications and self-efficacy. Among the 175 participants (70 in the intervention group and 105 in the control), results indicated a significantly greater reduction in direct medical costs over six months in the intervention group (USD 70.51) compared to the control group (USD 47.66), with medication cost being the primary cost driver (*p* < 0.001). The study’s limitations include its relatively short follow-up period and limited generalizability beyond Singapore [110].

## 5. Pharmacists in Diabetes Prevention

The role of pharmacists in diabetes prevention is vital, as they can provide awareness, screening, and patient direction toward beneficial lifestyle changes. Diabetes prevention includes implementing education, risk assessment, and patient initiatives to promote behavioral changes in patients’ lives. Pharmacists can leverage screening tools, such as the ADA diabetes risk test, to help identify patients at a higher risk of developing T2D [111].

Specific populations are more susceptible to diabetes due to genetic predisposition, lifestyle choices, and socioeconomic factors. Populations considered at higher risk for diabetes include those who are overweight or obese, sedentary, or aged 45 years and older, or those who belong to higher-risk ethnic groups, including African American, Hispanic/Latino, American Indian, and Alaskan Native communities. Pharmacists may address disparities in diabetes risk by offering culturally appropriate counseling and facilitating access to preventive services [112].

Beyond risk assessment, pharmacists can assist patients in implementing lifestyle intervention approaches such as those outlined in the CDC’s National Diabetes Prevention Program (DPP), which incorporates diet, physical activity, and behavioral counseling. Pharmacists can encourage patients to establish realistic goals, such as achieving at least 150 min/week of moderate-intensity aerobic exercise, reducing processed sugar intake, and minimizing stress as much as possible. All of these factors have been linked to a lower risk of developing future T2D [112].

In addition, community pharmacists may have the unique opportunity to collaborate with physicians, dietitians, and other healthcare providers to develop individualized diabetes prevention strategies. Proactive engagement by pharmacists may support early interventions aimed at delaying or potentially preventing the onset of diabetes.

## 6. Efficacy of Pharmacist Intervention

The efficacy of pharmacist-delivered interventions in managing diabetes has been extensively studied. Significant evidence demonstrates the positive impact of these interventions on patient outcomes, complications, and healthcare costs. Pharmacists, as experts in medication and accessible healthcare providers, are uniquely positioned to educate patients, optimize therapies, and monitor adherence to treatment regimens.

### 6.1. Local Pharmacist Interventions

Pharmacist-led interventions in patient education, critical medication evaluation, outcome assessments, and provider recommendations can enhance glycemic control in adults with T2D, as reported in a 2022 meta-analysis. This meta-analysis further demonstrated that these interventions have helped reduce HbA1c, especially in T2D patients with higher baseline HbA1c levels, whereas usual care does not yield the same reduction in HbA1c [103]. Additionally, another meta-analysis reported that interactive learning and patient-centered methods can improve pharmacists’ knowledge, skills, and attitudes for managing chronic conditions, ultimately improving chronic disease management [113].

Several critical pharmacist interventions have been shown to enhance diabetes management, including but not limited to the following:

Medication therapy management (MTM): Pharmacists assist in modifying medication regimens to enhance glycemic control and lower side effects. *Patient education* provides culturally competent education and counseling on self-care, medication adherence, and blood glucose monitoring. Continuous glucose monitoring (CGM) and insulin titration support help patients analyze CGM data and adjust insulin therapy accordingly. Lifestyle modification coaching and preventive care support patient efforts in active lifestyle changes to prevent the progression of diabetes and health complications. In addition to traditional community pharmacy settings, telepharmacy and digital health have offered pharmacists increased access to patient-centered care, including remote patient monitoring and virtual care for diabetes education and self-care. This innovative approach has improved access to care for rural and underserved populations while reducing clinical care disparities [113].

**Figure 2:**
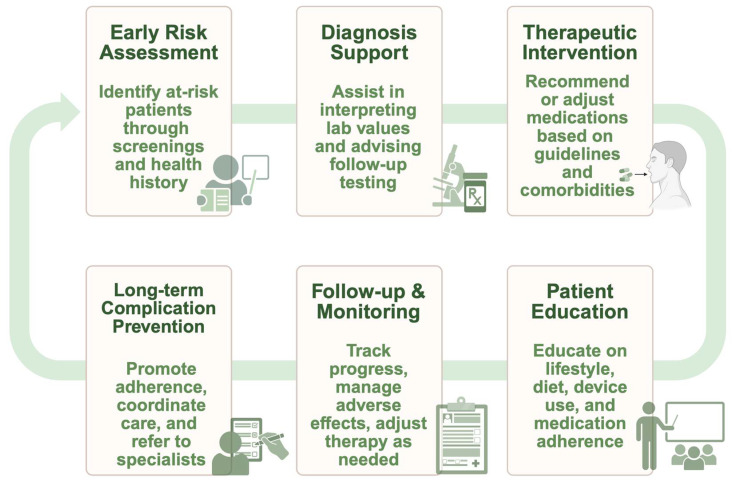
Timeline: pharmacist-led diabetes care trajectory. This timeline illustrates the pharmacist’s scope of care in managing T2D. It encompasses early risk assessment and diagnostic support through pharmacist participation, which involves identifying at-risk individuals, interpreting laboratory values, and recommending follow-up diagnostic assessments. This extends to clinical interventions, including their recommendations or dose adjustments for medications while considering comorbidities and clinical guidelines, as well as patient education on lifestyle changes, medication use, and device training to ensure adherence to pharmacotherapy. Monitoring and follow-up include therapy modifications and the management of adverse events, while long-term complications primarily depend on adherence, effective care coordination, and timely consultations with specialists. This figure was created in BioRender by Alshahrouri, B. (www.biorender.com/j553ie7, May 2025), a graphic design platform.

As healthcare systems prioritize person-centered and interprofessional care, pharmacists will remain vital members of the diabetes treatment team tasked with optimizing diabetes management therapy. Increasing pharmacist-led initiatives to manage diabetes will lead to improved glycemic control, fewer hospitalizations, and enhanced patient quality of life. Figure 2 provides a visual overview of the pharmacist-led care trajectory in diabetes management, highlighting key intervention points across the continuum, from early risk identification to the prevention of long-term complications. According to a recent systematic review, pharmacist-led interventions significantly improved clinical outcomes in T2D patients, reducing HbA1c by 0.70%, LDL cholesterol by 5.51 mg/dL, systolic BP by 4.58 mmHg, diastolic BP by 1.90 mmHg, BMI by 0.47 kg/m^2^, and fasting blood glucose by 19.82 mg/dL, and significantly enhancing medication adherence [114].

### 6.2. Global- and System-Level Integration of Pharmacist Roles

Pharmacist-led diabetes care models can be adapted in various healthcare systems; however, their uptake relies on the policies of individual national governments, the scope of practice regulations for pharmacists, and the existing reimbursement structures. Internationally, the number of clinical roles for pharmacists has been steadily increasing. The United Kingdom and Canada have incorporated pharmacists into primary care networks, allowing for medication reviews, chronic disease education, and, in some sites, independent prescribing. In Australia, pharmacists participate in structured diabetes screening and management programs, often collaborating with general practitioners [115]. In the U.S., pharmacists play a critical role in bridging care gaps, especially in underserved or rural areas; however, the expansion of their clinical role is hindered by state-imposed restrictions and the absence of federal provider status. Adding pharmacy billing pathways through Medicare, collaborative practice agreements (CPAs), and digital strategies such as telepharmacy and AI predictive risk assessments could facilitate the expansion of pharmacists’ care capacities [116]. Embracing lessons from international models while exploring feasible ways to align U.S. health policies to increase pharmacists’ involvement in diabetes prevention, adherence support, and long-term disease management is an area of growing interest.

Beyond the models implemented in the United States, the United Kingdom, Canada, and Australia, other nations have begun integrating pharmacists into diabetes care in diverse ways. Germany, through a pharmacist-led intensive care model in community pharmacies (the GLICEMIA 2.0 trial), was able to significantly improve glycemic control (reduced HbA1c by ~0.7% over one year) in patients with type 2 diabetes as compared to usual care [117]. Japan launched a pilot program where pharmacists and onsite dietitians in community pharmacies collaborated, improving HbA1c levels and dietary self-management of patients, indicating the advantage of a collaborative multidisciplinary approach in diabetes care [118]. In Brazil, community pharmacists in public pharmacies utilized point-of-care HbA1c screening and identified a significant number of patients who had previously undiagnosed prediabetes and diabetes and successfully referred and intervened in a timely manner [119]. Clinical pharmacists in India conducted medication reviews and patient counseling at hospital diabetes clinics, resulting in interventions that optimized therapy and increased safety for patients with diabetes and concomitant hypertension over five years [120]. In Sudan, a diabetic education program led by clinical pharmacists improved patients’ knowledge and attitudes about diabetes management in a tertiary diabetes clinic [121]. These examples from around the world reflect the increasing integration of pharmacists in teams for diabetes prevention and management, from screening to education, and direct patient care to therapy optimization.

### 6.3. U.S. Policy Frameworks Supporting Expanded Pharmacist Roles

Policy Frameworks Expanding Pharmacist Practice in Diabetes Care (2023–2025): Many recent U.S. federal and state policy efforts aim to expand pharmacists’ roles in diabetes care. Federal activities focus on credentialing pharmacists as recognized providers by legislation like the proposed Pharmacy and Medically Underserved Areas Enhancement Act, which would provide Medicare Part B reimbursement for clinical services from pharmacists—including chronic disease management for conditions such as T2D [122]. Providers’ status efforts involve a significant push from pharmacy organizations; for example, the APhA and ASHP supported introducing the above bill in 2023 to expand access and provide better outcomes for underserved populations [123]. There have also been new activities happening at the state level, with most states modifying laws or regulations to give authority and payment to pharmacists, primarily because of the publicly available evidence of pharmacists providing diabetes care. By 2023, 43 state Medicaid programs recognized pharmacists as providers, and all 50 states allow for collaborative practice agreements (CPAs) that would enable pharmacists to initiate, change, or manage a patient’s drug therapy and diabetes, and other chronic conditions, under the supervision of a physician [123,124]. Several states (e.g., WY, VA, MD) also passed recent laws that require Medicaid, and even private insurers, to reimburse pharmacists for care provided to patients under CPAs and other applications of their scope of practice, allowing pharmacists to be compensated for services provided in their clinical role [122]. Large pharmacy associations such as the APhA and ASHP continue to take on advocacy roles at both the federal and state levels concerning reimbursement changes, and provider status, arguing that pharmacists will improve access to care in diabetes prevention and management and improve glycemic outcomes [123]. In contrast, the American Medical Association (AMA) has tried to hinder the effort to provide independent clinical authority to pharmacists. It has opposed federal legislation seeking to expand pharmacist services, expressing concerns for patient safety and arguing that this effort constitutes “scope creep” to the detriment of pharmacists, expanding unnecessary workload and fragmenting care [125]. In summary, from 2023 to 2025, a similar trend, or shift, has been noticed where pharmacists are moving toward an independent role in diabetes care through legislative changes, regulations, and advocacy activities through formalized collaborative practice agreements and payment for direct clinical services and recognition as a part of the healthcare team [122,124].

## 7. Conclusions and Future Remarks

Pharmacists are uniquely positioned to contribute to T2D prevention, screening, and management through patient education, medication optimization, and early intervention strategies. To maximize their impact, policies should support expanded pharmacist roles in interdisciplinary care teams, reimbursement for clinical services, and the integration of digital tools for patient outreach. For instance, AI-driven digital platforms have been utilized for electronic health records (EHRs) to identify patients at high risk of developing diabetes or experiencing its complications. These applications provided risk-stratification scores, enabling the pharmacist to prioritize and initiate risk-reduction interventions (such as medication therapy changes, lifestyle counseling, and patient education) promptly.

During recent pilot programs involving telepharmacy, pharmacists provided clinical, remote medication consultations, follow-ups, counseling, and monitoring adherence through telecommunication (video calls, mobile health apps, etc.). These technologies enable pharmacists to expand their access to pharmaceutical care in rural and medically underserved populations while decreasing HbA1c levels.

Strengthening pharmacist involvement in diabetes care can enhance outcomes, reduce health system liabilities, and promote more equitable access to chronic disease management [114]. Collaboration among pharmacists, physicians, and other healthcare providers will be essential for improving diabetes management and addressing the overall disease burden as a global strategy to combat the rise in diabetes in populations worldwide across all ages.

Diabetes management will likely involve advancements in smart insulin, personalized medicine, beta cell regeneration, and increased use of digital health through artificial intelligence. Pharmacists must be among the first to learn about new treatment modalities as they emerge, enabling them to provide evidence-based therapy to their patients.

## Figures and Tables

**Table 1:** Blood glucose-specific diagnostic tools for T2D.

Treatment Type	Description	Purpose	Advantages	Limitations
HbA1c Test	Measures average blood glucose levels over the past 2–3 months	Identify prediabetes or diabetes	No fasting required; reflects long-term control	Less reliable in some conditions (e.g., anemia)
Fasting Plasma Glucose (FPG)	Measures blood sugar after an overnight fast	Assess fasting glucose levels	Widely available and inexpensive	Requires fasting; may miss post-meal hyperglycemia
Oral Glucose Tolerance Test (OGTT)	Measures blood sugar before and after consuming a glucose-rich drink	Detect glucose intolerance	Highly sensitive for diagnosing prediabetes	Time-consuming; requires fasting and preparation
Random Plasma Glucose Test	Measures blood sugar levels at any time, regardless of fasting	Quick assessment of glucose levels	Useful in emergency settings	Not specific without symptoms; needs confirmation
Home Glucose Monitoring	Patients measure glucose levels using portable glucometers	Early detection of irregular patterns	Convenient for tracking over time	Relies on patient compliance and technique
Continuous Glucose Monitoring (CGM)	Monitors glucose levels continuously via a wearable device	Detects trends and fluctuations	Provides detailed glucose trends	Expensive; not widely used for pre-diagnosis

**Table 2:** Risk indicators and monitoring tools for T2D.

Tool	Description	Purpose	Advantages	Limitations
BMI and Waist Circumference	Assesses obesity-related risk factors	Identifies individuals at higher risk	Simple and non-invasive	Does not directly measure glucose metabolism
Risk Assessment Questionnaires	Tools like the ADA Risk Test assess lifestyle and genetic factors	Identifies high-risk individuals	Quick and easy to administer	Self-reported data may not be accurate
Lipid Panel	Measures cholesterol and triglyceride levels	Evaluates metabolic syndrome	Provides insight into related risk factors	Indirect indicator of diabetes risk
C-Reactive Protein (CRP)	Measures inflammation levels linked to insulin resistance	Detects inflammation-related risks	Adds insight into cardiovascular risk	Not specific to diabetes alone

The provided tables summarize standardized tools for diagnosing T2D, including examples and descriptions. The aim is to identify early signs of abnormal glucose metabolism to allow for timely intervention.

## Data Availability

Not applicable.
